# Back massage as an approach to reduce labor anxiety in primigravida mothers

**DOI:** 10.6026/973206300213502

**Published:** 2025-10-31

**Authors:** Soma Bepari, Siva Subramanian N., Mahalakshmi B.

**Affiliations:** 1 Department of Obstetrics & Gynecological Nursing, Nootan College of Nursing, Sankalchand Patel University, Visnagar, Gujarat - 384315, India; 2 Department of Psychiatric Nursing, Nootan College of Nursing, Sankalchand Patel University, Visnagar, Gujarat - 384315, India; 3 Department of Paediatric Nursing, Nootan College of Nursing, Sankalchand Patel University, Visnagar ,Gujarat - 384315, India

**Keywords:** Effleurage massage, labor anxiety, labor duration, primigravida mothers, non-pharmacological intervention, intrapartum care, childbirth

## Abstract

Effleurage massage is a gentle, rhythmic technique that may promote relaxation and reduce psychological distress during labor is of interest. Therefore, it is of interest to assess the effectiveness of effleurage back massage on reducing anxiety and labor duration among 180 primigravida mothers in Bharuch, Gujarat. The findings showed a significant reduction in anxiety scores and shorter mean labor duration in the experimental group (p < 0.001). Data shows that effleurage massage is a simple, effective intervention to improve psychological well-being and labor outcomes. It may be recommended as part of intrapartum nursing care for first-time mothers.

## Background:

Childbirth is recognized universally as a profound, life-altering experience that encompasses significant physical and emotional dimensions, particularly for primigravida mothers [1]. Anxiety during labor can greatly impact maternal outcomes, affecting pain perception, emotional well-being and physiological progression of childbirth [2]. Elevated anxiety levels are linked with increased secretion of stress hormones such as cortisol and catecholamines, resulting in decreased uterine blood flow, ineffective contractions and prolonged labor [2]. The close interplay between psychological and physiological states highlights the need for effective maternal anxiety management to promote positive birth experiences [3]. Pharmacological pain relief methods, such as epidural analgesia and systemic opioids, though effective for pain, show limited efficacy in reducing labor anxiety and carry risks like prolonged labor, maternal hypotension and adverse neonatal effects [4]. Resource limitations and cultural preferences in developing countries further restrict pharmacological analgesia use [5]. Consequently, clinical focus has shifted toward non-pharmacological interventions that are safe, economical and culturally acceptable [5]. Effleurage massage, characterized by rhythmic, gentle stroking of the skin, has emerged as a promising technique for reducing maternal anxiety by stimulating cutaneous mechanoreceptors, enhancing endogenous opioid production and activating the parasympathetic nervous system [6]. This approach also supports emotional bonding, reduces fear of childbirth and enhances maternal satisfaction [7, 8]. Experimental research by Mortazavi *et al.* (2012) demonstrated significantly lower anxiety scores in mothers receiving massage during labor compared with standard care [9]. Gallo *et al.* (2013) similarly reported reduced psychological distress and improved maternal satisfaction following therapeutic massage [10]. Maghalian *et al.* (2022) observed marked reductions in perceived labor stress after massage, reinforcing its psychological benefits [11]. Massage also appears to facilitate labor progression by promoting relaxation, enhancing uterine contractility and improving cervical dilation [12]. Bolbol-Haghighi *et al.* (2016) reported a significantly shorter active phase of labor in women receiving massage therapy [13]. Despite strong international evidence, Indian data-particularly from Gujarat-remains limited [14]. Regional studies by Vijayalakshmi *et al.* (2023) demonstrated promising results, confirming the effectiveness of massage in reducing anxiety and shortening labor within the Indian healthcare context [14]. Therefore, it is of interest to evaluate the effectiveness of effleurage back massage as a targeted non-pharmacological intervention for anxiety reduction and labor duration shortening among primigravida mothers admitted to selected hospitals in Bharuch, Gujarat.

## Methodology:

## Research design and setting:

This study adopted a quasi-experimental pre-test post-test control group design and study setting conducted in the labor rooms of selected tertiary care hospitals in Bharuch district, Gujarat, where normal deliveries are routinely conducted and where supportive non-pharmacological intervention could be administered during labor without interrupting clinical procedures.

## Sample and sampling technique:

A total of 180 primigravida mothers in the active stage of labor were enrolled using a non-probability convenience sampling technique, considering feasibility constraints. Participants were then randomly allocated into experimental (n=90) and control (n=90) groups.

## Inclusion and exclusion criteria:

Inclusion criteria comprised primigravida women in active labor, aged 18-35 years, who could communicate in Gujarati or Hindi and were willing to give informed consent. Women with high-risk pregnancies, diagnosed psychiatric disorders, or complications during labor (e.g., fetal distress, eclampsia) were excluded. Additionally, women who were unavailable or unwilling to participate at the time of intervention were also excluded.

## Intervention and tools:

The experimental group received effleurage back massage using light, rhythmic stroking over the lumbar region during uterine contractions at intervals throughout the active phase of labor. The control group received routine nursing care without any intervention. Anxiety was measured using the Visual Facial Anxiety Scale and labor duration was recorded using the partograph from cervical dilation of 4 cm to full dilation (10 cm). A structured socio-demographic data sheet was also used to collect participant background information.

## Data collection and statistical analysis:

Data collection was carried out by trained nursing personnel under supervision. Pain and anxiety scores were recorded before and after the intervention in both groups and total duration of labor were extracted from the partograph. Data were analyzed using Statistical Package for the Social Sciences (SPSS) software, employing descriptive statistics (mean, SD, percentage) and inferential tests, including paired and unpaired t-tests and chi-square tests to evaluate group differences. A p-value of <0.05 was considered statistically significant for all analyses.

## Results:

As shown in Table 1 (see PDF) and Figure 1 (see PDF) the mean anxiety score in the experimental group significantly decreased from 6.51 ± 1.11 to 5.51 ± 1.09 following the effleurage massage intervention (p < 0.001). In contrast, the control group showed no significant change in anxiety scores (p = 0.180). This highlights the psychological benefit of the massage intervention. As summarized in Table 2 (see PDF), the association analysis revealed that education level and residence were significantly associated with pre-test anxiety in the experimental group, while no demographic variables were significantly associated in the control group. This suggests that individual background may influence initial anxiety levels and receptiveness to interventions such as massage.

## Discussion:

The findings from this study clearly demonstrate the efficacy of effleurage back massage as a non-pharmacological intervention for reducing labor anxiety and shortening labor duration among primigravida mothers. Our results indicated a statistically significant decrease in anxiety levels post-intervention in the experimental group (p<0.001), consistent with other studies that examined massage as a relaxation method during labor. For instance, Pawale and Salunkhe (2020) similarly reported a significant reduction in anxiety scores following back massage among first-time mothers [15]. This aligns well with the systematic review by Choudhary *et al.* (2021), which concluded massage therapy effectively reduces anxiety associated with childbirth [16]. The observed reduction in anxiety is explained by physiological theories, particularly gate-control theory, where gentle touch stimuli provided through massage block or diminish painful sensations, subsequently lowering anxiety [17]. Similarly, Field *et al.* (2005) emphasizes the role of massage in promoting parasympathetic activity, reducing cortisol and enhancing serotonin and dopamine levels, directly mitigating anxiety [18]. A randomized controlled trial by Chang *et al.* (2002) conducted in Taiwan further supports our findings, reporting a notable anxiety reduction among women receiving massage compared to routine care [19]. Our study also noted a substantial shortening in the duration of labor in mothers receiving massage (mean duration: 8.03 hours) compared to the control group (12.35 hours, p<0.001). Another randomized trial by Yuksel *et al.* (2017) observed reduced labor duration attributed to non-pharmacological interventions, particularly massage and breathing exercises [20]. The beneficial role of massage therapy in improving uterine blood flow and contraction efficiency, leading to reduced labor duration, has been extensively reported in literature [21].

Our demographic association analysis identified significant correlations between educational status and residence with initial anxiety levels. These findings resonate with previous research, such as Anim-Somuah *et al.* (2018), who highlighted that educational status significantly impacts maternal anxiety and response to labor interventions [22]. Similarly, Smith *et al.* (2018), in their comprehensive Cochrane review, acknowledged that maternal anxiety levels and comfort with labor interventions could vary with demographic backgrounds such as education and socioeconomic status [23]. The effectiveness of massage as a labor intervention has received consistent support across global studies. A clinical trial by Norhapifah demonstrated significant reductions in anxiety and pain among primigravida mothers following back massage [24]. These outcomes collectively support massage as an accessible and effective method for reducing childbirth anxiety. Similarly, Czech *et al.* (2018) and Thomson *et al.* (2019) independently reported positive outcomes from manual therapies, further substantiating massage as a beneficial intrapartum intervention [25, 27]. These findings align with previous research indicating that lower educational attainment may correlate with limited childbirth preparedness, higher anxiety and fear during labor, possibly due to decreased awareness of labor procedures and pain management options [22]. Furthermore, a study by Boateng *et al.* (2019) similarly found that maternal anxiety varied significantly based on residential status, with rural women displaying greater anxiety possibly due to limited access to antenatal education and fewer labor support interventions available in rural healthcare facilities [26]. In contrast, other demographic variables-such as age, type of family, occupation and in come-were found to significantly influence anxiety scores in our study. The absence of significant associations with these variables echoes previous findings from a systematic review by Thomson *et al.* (2019), suggesting that demographic variables like family type and occupational status typically have minimal direct impact on anxiety perception during labor [27]. These insights highlight the necessity of targeted educational and psychosocial support interventions, particularly for lower-educated and rural primigravida mothers, to effectively reduce anxiety and optimize childbirth experiences. Non-pharmacological strategies like massage also play a crucial role in maternal satisfaction and labor experience, as reported by Boateng *et al.* (2019), who found that midwives and nurses viewed massage as a valuable, patient-preferred alternative to medication [26]. These findings are further supported by Moncy *et al.* (2024), who reported that back massage during labour significantly reduced analgesic use, pain intensity, and labour duration among primigravida mothers [28]. Likewise, Weljale (2021) found that back massage effectively relieved labour pain and anxiety, confirming it as a simple, safe, and beneficial non-pharmacological intervention [29]. Overall, our findings significantly add to existing evidence advocating for effleurage massage as a safe, cost-effective and culturally acceptable method to alleviate labor anxiety and shorten labor duration. Given these consistent positive outcomes, healthcare systems should strongly consider integrating such non-pharmacological interventions into routine maternity care protocols to improve maternal experiences during childbirth.
